# An exploration of the factors that influence footwear selection in women with Rheumatoid Arthritis – a qualitative study

**DOI:** 10.1186/1757-1146-3-S1-O18

**Published:** 2010-12-20

**Authors:** Serena Naidoo, Stephanie Anderson, Joanna Mills, Stephanie Parsons, Stephanie Breeden, Emma Bevan, Camilla Edwards, Simon Otter

**Affiliations:** 1 University of Brighton, Brighton, UK

## Background

Studies report that women with Rheumatoid Arthritis (RA) are not wearing therapeutic footwear. It is likely they are wearing normal retail footwear. Previous research gives limited information on people’s perceptions on the relationships that exist between retail footwear, well-being and quality of life (QOL). This study aimed to explore these perceptions and to identify the factors influencing footwear selection.

## Methods

Eleven women with RA wearing retail footwear were recruited from an out-patient podiatry clinic in the south east of England. Semi-structured interviews were carried out and a hermeneutical approach; interpretative phenomenological analysis of transcripts occurred to identify recurring themes.

## Results

Six main themes were revealed from the analysis. These included: (1) the nature of foot complaints and deformities, (2) aesthetic appearance and design of footwear, (3) body image, (4) psychosocial aspects, (5) Perceptions of footwear and (6) the therapeutic value of retail shoes.

## Conclusions

Retail footwear for these women with RA has impacted on their individuality which has been shown to link significantly with their body images. Whilst footwear itself has not impacted solely on the well-being of these women; the disease process has. These consequences have been identified in their quality of life.

